# Continuous Splenogonadal Fusion Diagnosed by Laparoscopy in a Child With a Communicating Hydrocele

**DOI:** 10.7759/cureus.67836

**Published:** 2024-08-26

**Authors:** Claudia Berrondo

**Affiliations:** 1 Surgery/Pediatric Urology, University of Nebraska Medical Center, Omaha, USA; 2 Pediatric Urology, Children's Nebraska, Omaha, USA

**Keywords:** inguinal hernia, communicating hydrocele, laparoscopy, continuous splenogonadal fusion, splenogonadal fusion

## Abstract

Splenogonadal fusion is a rare congenital anomaly primarily affecting males, characterized by an abnormal fusion of the spleen and the gonad. There are two primary forms: continuous, in which the normal spleen is directly connected to the gonad via a cord of fibrous or splenic tissue, or a combination of both, and discontinuous, in which ectopic splenic tissue fuses to the gonad without connection to the normal spleen. Continuous splenogonadal fusion is often associated with other congenital defects, such as cryptorchidism, limb anomalies, and micrognathia. Due to its rarity and nonspecific symptoms, splenogonadal fusion is typically diagnosed incidentally during surgery for undescended testis or inguinal hernia. We present a case of a five-year-old boy with a communicating hydrocele who underwent surgical repair. Intraoperative findings during inguinal exploration revealed a fibrous connection to the upper pole of the left testicle, extending into the internal inguinal ring. Diagnostic laparoscopy confirmed continuous splenogonadal fusion, demonstrating splenic tissue and a fibrous cord with islands of splenic tissue extending from the spleen to the internal inguinal ring. A portion of the fibrous cord with splenic nodules was excised and examined pathologically, confirming the diagnosis of splenogonadal fusion. The diagnosis of continuous splenogonadal fusion primarily relies on intraoperative findings during surgery for other conditions. Laparoscopy can be instrumental in diagnosing this rare condition.

## Introduction

Splenogonadal fusion is a rare congenital anomaly. Incidence is more common in males, and the anomaly is most often diagnosed during exploration for either an inguinal hernia or an undescended testicle. It may also present as a mass, masquerading as testicular cancer [1-3].

There are two types of splenogonadal fusion: continuous and discontinuous. The continuous form, in which the normal and orthotopic spleen and gonad are connected by a cord of splenic tissue or fibrous cord, is the most common [1-4]. The cord may consist of pure splenic tissue, splenic nodules connected by a fibrous cord, or a purely fibrous cord. The cord may take an intraperitoneal or retroperitoneal path to the gonad. The continuous form is frequently associated with other congenital abnormalities, most commonly cryptorchidism, limb defects, and micrognathia. The discontinuous form consists of the fusion of an ectopic or accessory spleen to the gonad with no attachment to the normal, orthotopic spleen. The discontinuous form is usually found in isolation with no other associated congenital anomalies [1,2,4].

We report a patient with continuous splenogonadal fusion identified during surgery for a communicating hydrocele. Laparoscopy confirmed the diagnosis, highlighting its valuable role in uncovering this rare anomaly.

## Case presentation

A five-year-old Hispanic male presented to the pediatric urology clinic with a three-year history of left scrotal swelling. The swelling was painless and intermittent. There was no history of trauma, and there were no associated erythema or urinary symptoms. The patient had no significant medical or surgical history, and his family history was unremarkable.

A physical examination revealed a non-tender, left-sided transilluminating scrotal mass that reduced easily with gentle pressure. The left testicle was palpable, normal in size and consistency, and without evidence of associated masses. The right testicle and scrotum were normal. The remainder of his physical exam was unremarkable.

Given the clinical presentation consistent with a communicating hydrocele, the patient underwent elective surgical repair. During inguinal exploration, the hernia sac was identified and isolated. Within the hernia sac, a firm, cord-like structure was identified and found to be extending from the upper pole of the left testis.

**Figure 1:**
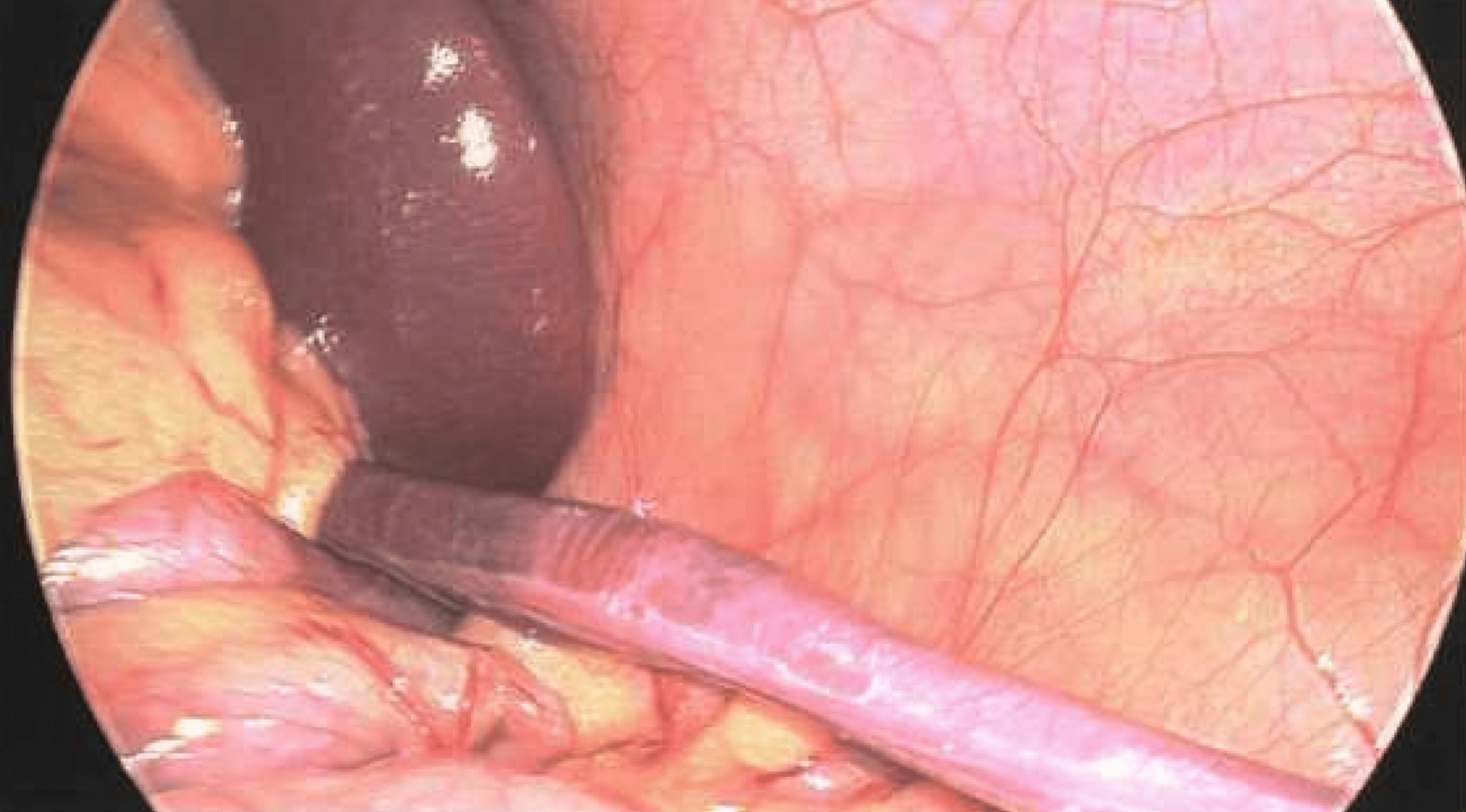
Laparoscopic view of the orthotopic spleen with a tongue-like extension of splenic tissue projecting inferiorly.

**Figure 2:**
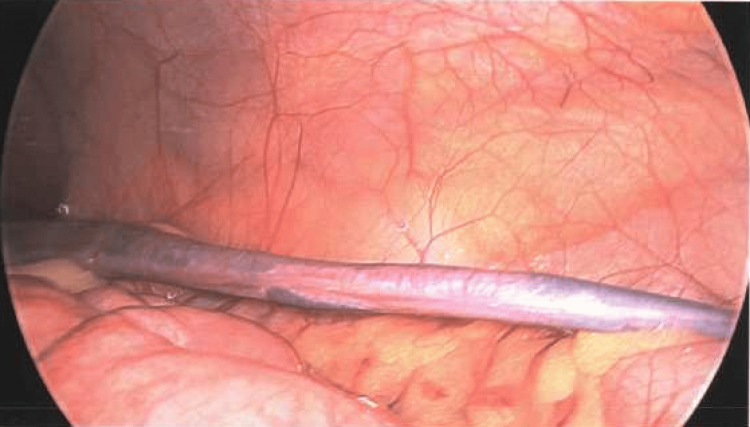
Laparoscopic view of the splenogonadal tissue extending transperitoneally through the abdominal cavity.

**Figure 3:**
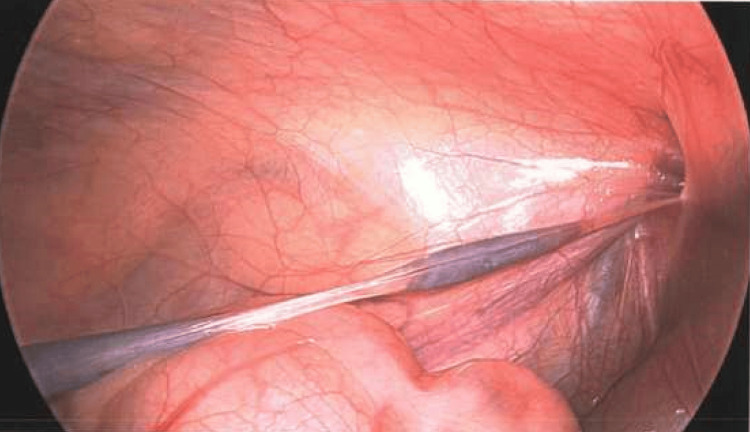
Laparoscopic view of the splenogonadal fusion tissue extending into the left internal ring.

To further delineate the extent of the anomalous structure, diagnostic laparoscopy was performed. Laparoscopically, the continuous form of splenogonadal fusion was confirmed, with a fibrous cord containing splenic nodules extending from the upper pole of the spleen to the left internal inguinal ring (Figures 1-3).

**Figure 4:**
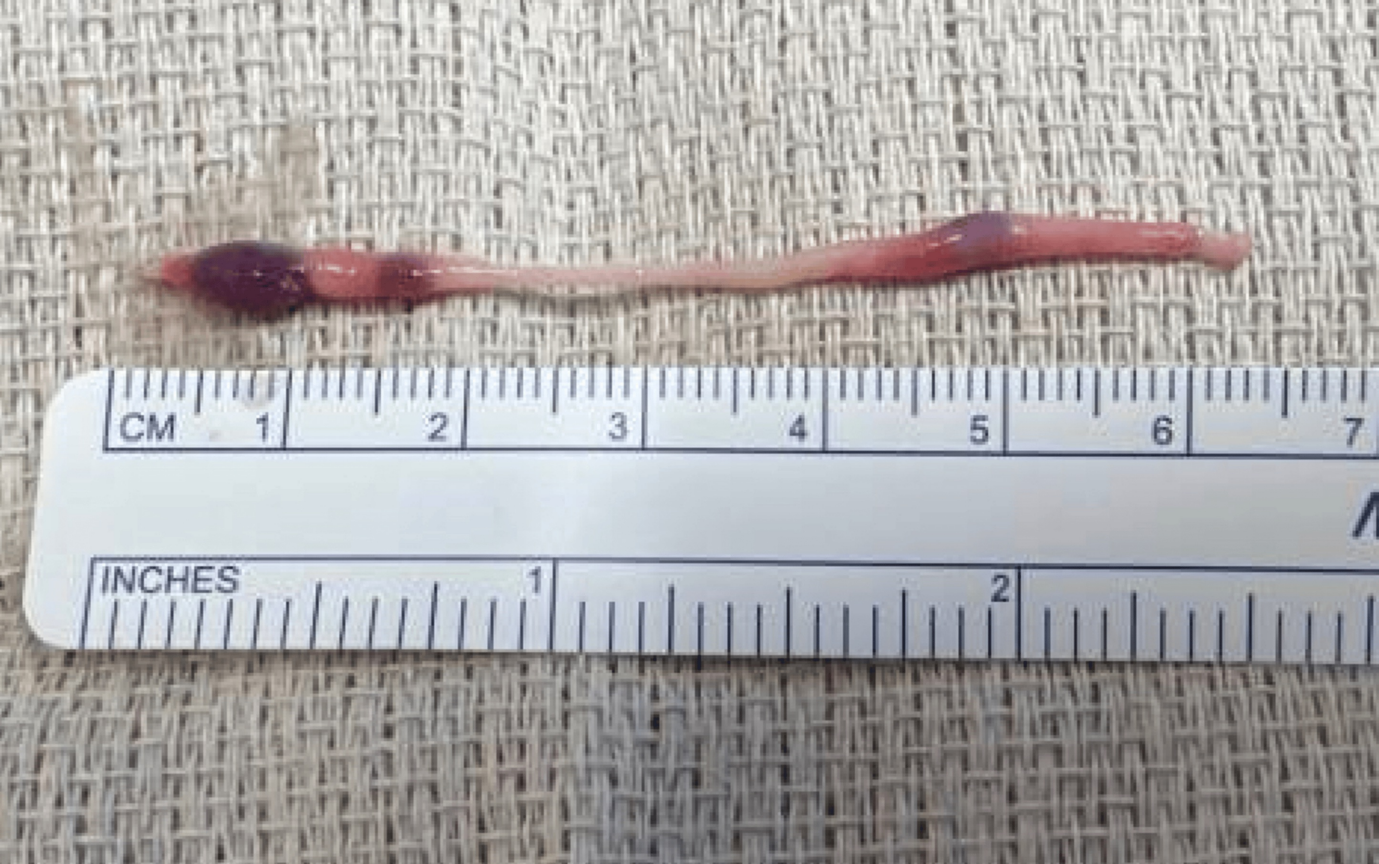
A portion of a fibrous cord containing nodules of splenic tissue excised from within the hernia sac. The distal aspect of the cord (right side of the figure) was surgically divided from the superior pole of the testicle. The proximal portion of the cord (left side of the figure) extended from the abdominal cavity through the internal inguinal ring.

There were no other associated congenital anomalies aside from the hernia. The distal portion of the cord was divided from the testis, and a segment was excised for histopathological examination (Figure 4).

Pathologic examination of the excised tissue confirmed the presence of splenic tissue within the fibrous stroma. The patient recovered uneventfully and was discharged home postoperatively. At the four-week postoperative follow-up appointment, the patient was asymptomatic without evidence of recurrence of the hydrocele.

## Discussion

Splenogonadal fusion is a rare, benign congenital anomaly primarily affecting males. Due to the internal location of the female gonads, reported cases in females are scarce, and the incidence is likely underestimated. The exact incidence in females is unknown, but the male-to-female ratio is approximately 16:1 [2-5]. Typically diagnosed in infancy or childhood, this condition often presents incidentally during surgical exploration for inguinal hernia, undescended testicles, or a suspicious inguinal or testicular mass. While mostly asymptomatic, some patients develop an asymptomatic scrotal mass, raising concern for malignancy. Patients may also present with symptoms related to the splenic tissue, such as leukemia, malaria, mononucleosis, or splenic rupture. Rarely, splenogonadal fusion can lead to intestinal obstruction from an intra-abdominal cord [1,2,5,6].

Splenogonadal fusion has been categorized into two separate forms: continuous, in which the normal and orthotopic spleen and gonad are connected, accounting for 55% of cases, and discontinuous, in which ectopic splenic tissue is fused to the gonad with no connection to the normal and orthotopic spleen, accounting for 45% of cases [1,2,7-9].

In the continuous form, the orthotopic spleen is connected to the gonad with either continuous splenic tissue, a purely fibrous cord, or a combination of splenic tissue and fibrous cord, which may include nodules of splenic tissue separated by a fibrous cord. The connection typically originates in the upper pole of the spleen and terminates in the upper pole of the testicle. The cord may take a retroperitoneal or intraperitoneal course [1,2,7-9].

Associated congenital anomalies are more common in the continuous type compared to the discontinuous type. The continuous form may also be associated with other congenital anomalies up to 50% of the time, with the most common anomalies being cryptorchidism, inguinal hernia (approximately 30% of the time), limb anomalies, and micrognathia. Other less common associated anomalies have also been reported, including cardiac anomalies, cleft palate, Potter syndrome, hypospadias, disorders of sexual differentiation, and spina bifida [1,2,7-11].

In the discontinuous form, there is no connection between the normal spleen and the ectopic splenic tissue. The ectopic splenic tissue may be adherent to the spermatic cord or testicle or fused to the gonad, typically the upper pole of the testicle. The splenic tissue may be inseparable from the gonad [1,2,7-10,12].

The exact cause of splenogonadal fusion remains unclear. The leading theory suggests that it occurs early during development, prior to testicular descent, at five to six weeks gestation. During this time, the embryonic gut rotates around its axis, bringing the genital ridge and splenic outline into close proximity. The proximity of the structures is likely what leads to fusion, and it is almost exclusively found on the left side [1,2,5,7,10,13].

Diagnosing splenogonadal fusion preoperatively remains a challenge. In patients with associated intraabdominal testicles, the diagnosis is typically made during laparoscopy [5,7-9,11]. For the continuous form, suspicion generally arises during surgery for other conditions. In such cases, laparoscopy can also be a valuable tool for the definitive diagnosis and treatment [14,15].

## Conclusions

Splenogonadal fusion is a rare congenital anomaly primarily affecting males, characterized by the abnormal attachment of the spleen or splenic tissue to the gonad. This unique condition often presents as an incidental finding during surgical exploration for other conditions, such as inguinal hernia or an undescended testicle. The lack of specific preoperative symptoms contributes to the diagnostic challenge. While the exact etiology remains unknown, the prevailing hypothesis is that splenogonadal fusion arises due to the close proximity of the developing spleen and left gonad during embryogenesis. Laparoscopy can be a valuable tool for the diagnosis of splenogonadal fusion. This allows for minimally invasive and direct visualization of the abdominal cavity, enabling accurate identification of the anomaly. Furthermore, laparoscopic intervention can be curative, avoiding the need for more invasive exploration. Given the rarity of splenogonadal fusion, a high index of suspicion is needed for an accurate diagnosis of this condition.
